# Impact of Environmental Quality on Healthcare Expenditures in Developing Countries: A Panel Data Approach

**DOI:** 10.3390/healthcare10091608

**Published:** 2022-08-24

**Authors:** Asim Anwar, Shabir Hyder, Russell Bennett, Mustafa Younis

**Affiliations:** 1 Department of Management Sciences, COMSATS University Islamabad, Islamabad 43600, Pakistan; 2 Department of Health Policy and Management, School of Health Sciences, Jackson State University, Jackson, MS 39217, USA; 3 School of Business & Economics, University Putra Malaysia, Serdang 43400, Malaysia

**Keywords:** air pollution, temperature, health expenditures, generalized method of moment (GMM), developing countries

## Abstract

Objective: The deterioration in environmental quality has an economic and social cost. The aim of this study is to analyze the impact of environmental factors on health expenditures in developing countries. Method: To analyze the relationship between environmental quality (air pollution and temperature) and health expenditure in thirty-three developing countries, the study uses system generalized method of moments (GMM) using data from 2000 to 2017. Results: The results suggest a positive effect of both air pollution and temperature on health expenditure. However, the effect is highest for government health expenditure, followed by private and total health expenditure in the studied countries. The results further suggest that the impact of environmental factors is greater in higher-income countries when we divide the studied countries into two groups, i.e., higher- and lower-income countries. Conclusion: Our results are interesting and informative for the policy makers to design such policies to attain better environmental quality and social well-being. The increased healthcare expenditures due to increased air pollution and climate change necessitate for an efficient, reliable, affordable and modern energy policy by emphasizing the use of clean and renewable energy in these countries that ensure better health for the masses. Furthermore, a smart and sustainable environmentally friendly economic growth policy is necessary to ensure better health for the masses.

## 1. Introduction

Growing economic activities, along with an increase in energy consumption, leads to deterioration in environmental quality. The worsening environmental quality in terms of air pollution and severe weather conditions poses a great threat to human lives as almost 99% of the worlds’ population lives in areas with air quality levels that already exceed WHO limits [1,2,3].

Air pollution is considered to be a silent killer and termed as a major risk component for health in both infants and adults [4,5], lower labor supply and labor productivity [6,7,8]. Ref. [9] estimated air pollution was responsible for 24% of cases of stroke, 25% of ischemic heart disease, 28% of lung cancer and 43% of chronic obstructive respiratory disease. Ambient air pollution (PM_2.5_) was responsible for almost 4.2 million deaths in 2018, of which 18% were attributed to acute respiratory infection [4]. The most lethal pollutants closely associated with human health are PM_2.5_ [10]. Air pollution affects both developed and underdeveloped countries alike, but underdeveloped countries experience the highest burden of disease, especially in the Western Pacific and Southeast Asian regions [11].

Along with air pollution, the change in climate threatens human health through the weather we experience, air we breathe, water we drink and the food we eat [12]. Extreme temperature is associated with higher hospital admissions for cardiovascular, respiratory and kidney disorders [13,14]. Ref. [15] estimates almost 250,000 deaths annually due to climate change by 2030 through direct exposure to extreme weather conditions (e.g., heat waves) or indirectly through the spread of diseases, water inaccessibility and food insecurity [16].

The literature on the social cost of environmental factors (air pollution and temperature) mainly focuses on morbidity and mortality [2,17,18,19], contemporaneous and long-term costs [8,20,21,22]. However, the health-related risk associated with air pollution and global warming has important economic spillovers on individuals’ savings, medical expenses and healthcare demand at the aggregate level [16]. Limited studies have examined the health expenditures associated with environmental factors being an important part of its social cost [16,23].

Therefore, the purpose of this research is to contribute to the literature by studying the impact of environmental factors (air pollution and temperature) on health expenditures in developing countries. Secondly, previous studies have studied the association between air pollution and health spending using carbon monoxide (CO), carbon dioxide (CO_2_), nitrogen oxide (NOx) and sulfur dioxide (SO_2_) as measures of air pollution [24,25,26,27,28], whereas this study used PM_2.5_ as a measure of air pollution as it is considered to be a more useful indicator of air pollution. Thirdly, the studies examined the relationship between temperature and health expenditure using cross sectional data, while we use panel data to analyze the role of climate change in affecting health expenditure in developing countries.

## 2. Literature Review

The growing health expenditure has become an important concern for most underdeveloped countries [29]. In the literature, different studies have found various determinants of increases in health expenditure over time, such as income, aging population, technology, health facilities and services [30,31].

The deterioration of the environment through an increase in air pollution has not only short-term but also long-term effects on health and economic outcomes [6,8,16,20,21,22,23]. Literature on the long-term impact of environmental factors can be roughly divided into two strands. The first strand of literature studies the long-term impact of environmental factors over health and its related costs. For example, [8] found a long-term impact of forest fires on humans, i.e., the impact of forest fires in 1997 could even be felt after a decade. Ref. [22] showed the impact of clean air pollution legislation on the increase in earnings in adulthood because of a low exposure to pollutants in childhood. The second strand of literature showed the long-term impact of healthcare expenditure while accounting for environmental factors. For example, Fernandez (2017) showed the impact of various pollutants on healthcare expenditure; however, most of the effect on healthcare expenditure is attributed to its past values. This view is enforced by the findings of [24] where pollutants increase healthcare expenditure; however, the major contributor still seems to be the historical expenditure on health.

The World Trade Organization (WTO) estimates that after forty years, the global economic cost of ambient air pollution will amount to 1 percent of the total gross national product (GNP), where the related medical expenditure in the long-term will dominate. Ref. [32], in their study on 49 counties of Canada, found that counties with poor air quality had higher medical expenditure, while there was lower medical expenditure in counties with better air quality. Ref. [26], using carbon monoxide (CO), nitrogen oxide (NOx) and sulfur dioxide (SO_2_) as measures of air pollution for Organization of Economic Cooperation and Development (OECD) countries from 1980 to 1999, examined the short- and long-term impact of environmental quality on health care expenditure. Their results revealed a positive and significant association between air pollutants and health care expenditures. Ref. [24] studied the relationship between environmental quality (CO_2_ emissions) and health care expenditure disaggregated into (public health expenditure, national health expenditure and private health expenditure) using pooled OLS, fixed effect and system GMM methods for 15 Economic Community of West African States (ECOWAS) over the period of 1995 to 2014. Their results validated the positive and significant effect of CO_2_ emission on public and national health expenditure while no relationship was found in the case of private health expenditure. Ref. [28] studied the long-term relationship between environmental quality (CO_2_ emissions, carbon monoxide (CO), nitrogen oxide (NOx) and sulfur dioxide (SO_2_)) and health expenditure for 125 developing countries using panel co-integration from 1995 to 2012. The results suggest the existence of a long-term relationship between per capita health expenditure and environmental quality. The study suggests that CO_2_ has a strong explanatory power relative to other variables on health care expenditure.

[33] studied the impact of environmental quality (sulfur dioxide, carbon monoxide and carbon dioxide) and healthcare expenditure using ARDL approach over the period from 1967 to 2011. The study found that environmental quality significantly affects health expenditures in Iran. Ref. [34] examined the causal relationship between CO_2_ emissions, GDP growth and health spending using dynamic simultaneous equation models and GMM in 51 countries from the period 1995 to 2013. The study indicated a unidirectional causality from CO_2_ emissions to health expenditures in 51 studied countries.

Along with air pollution, climate change is also one of the greatest challenges the world is facing [35]. Ref. [36] states that average worlds’ temperature over the last 100 years has risen by 0.7 °C (1.3 °F) and will continue to increase in the future. The National Oceanic and Atmospheric Administration (NOAA) data reports 2020 was the second warmest year on record while Intergovernmental Panel on Climate Change (IPCC) reports that extreme hot, heat waves and hefty precipitation events will become more frequent [37,38]. The World Health Organization [39] said in its report that there is an increase of 125 million people who have been exposed to heat waves from 2000 to 2016. Any changes to seasonal average temperature can lead to increased illness and deaths [39]. The increase in average temperature can worsen chronic conditions such as respiratory, cerebrovascular disease, cardiovascular and diabetes-related conditions [39]. The studies in the literature have assessed the effect of extreme temperature on health and found a significant impact on human health [4,40]. Ref. [41] conducted a study to find the impact of average temperature, rainfall and humidity on climate sensitive infectious diseases, i.e., malaria, diarrheal disease, enteric fever, encephalitis, pneumonia and bacterial meningitis in adults. The results suggest a strong association between weather patterns and disease incidence in Bangladesh. Ref. [40] found a significant impact on human health through heat related diseases and thus increasing the burden on healthcare system in Perth, Australia. Ref. [42], in their study on extreme heat exposure and healthcare cost, found that extreme temperature caused a major economic burden on the healthcare system, especially for females, the elderly and low-income families, who have the highest healthcare cost. Although studies have examined the relationship between temperature and negative health outcomes, limited studies have estimated the health-related cost of temperature [40,42]. Therefore, the aim of this study is to find out the impact of air pollution and temperature on health expenditure in developing countries.

## 3. Model Specification and Data

The study follows the model initially proposed by [39], who studied the bi-variate relationship between health expenditure and income. The model proposed that it is the household income that drives healthcare expenditure. However, various studies expanded the model by including environmental factors such as [4,20,22]. The functional form of the model is given as:

*HEit = f(Xit, b) + eit* (1)

where *HEit* is the log of various measures of per capita healthcare expenditure, *Xit* represents the independent variables, *b* is the usual slope parameter and *e* is the error term with usual statistical characteristics, *i* denotes the country and *t* shows the year. Following the above-mentioned studies, we extend the basic model to include other explanatory variables, i.e., average annual temperature (*TEMP*), particulate matter (*PM_2.5_*) as a proxy for air pollution (mean annual exposure micrograms per cubic meter), gross domestic product per capita PPP, constant US dollars, secondary education level as percentage of gross school enrollment (*EDU*) and population density (*POP*). After including these variables, our econometric model can be written as:

*HE_it_ = β_0_ + β_1_ PM_2.5it_ + β_2_ TEMP_it_ + β_3_ GDP_it_ + β_4_ EDU_it_ + β_5_ POP_it_ + e_it_* (2)

The data is in log form to interpret the variables as elasticities. The study employed System Generalized Method of Moments (GMM) for the persistent memory of prior values of dependent variable [43,44,45]. As has been stated earlier, little literature exists that looks into the persistent impact of healthcare expenditure on its future values; therefore, this study will look into that impact using system GMM being a good method in this case. Such a method also coincides with the nature of data we have, i.e., the requirement for estimating such model is the number of cross sections to be greater than the time periods and this criterion is met for the system GMM as the number of cross sections is 33 while the time period is 18. Additionally, our model does not have more instruments than the number of cross sections [46]. Moreover, we also checked for the first and second order of error term’s serial correlation. A Hansen test was used to check for the orthogonality conditions. Moreover, for comparison and to check the robustness of our results, we also estimate the panel fixed effect model based on a Hausman test.

Data were obtained from the World Bank (World Development Indicators) for the period 2000 to 2018. For some countries, data were not fully available; therefore, our data set is unbalanced. Per capita healthcare expenditure and gross domestic product measured in Per Capita, PPP. The descriptive statistics for various variables are shown below in Table 1.

We investigated the correlation coefficients of the dependent variables with its lagged values, which are above the values of 0.8 (as a rule of thumb). Results are provided in Table 2.

## 4. Results

The results of the panel fixed effects model are shown in Table 3. We estimated the results for all three healthcare expenditures, i.e., government, private and total healthcare expenditures, as dependent variables. Five different specifications were estimated for each healthcare expenditure by adding each variable one by one.

Panel fixed effect model results shows that in most of the specifications, the environmental factors remained statistically insignificant, and this is especially true for the final, i.e., fifth specification, while other variables such as education, population density and income level are statistically significant.

The regression results using System Generalized Method of Moments (GMM) are shown in Table 4. This method provides results while controlling for the effect of potential endogeneity issue that could be raised because of unobserved heterogeneity and cross-sectional dependence. We ran the regression by adding variables one by one to control for their effects. The results are shown in Table 4.

We ran regression for three different dependent variables, i.e., firstly, the dependent variable is government health expenditure, followed by private health expenditure and total health expenditure, while in all three specifications, independent variables remain the same. All of our variables are in logarithmic form; therefore, all the coefficients are showing elasticities. In Table 3, our result in column 1 shows the effect of air pollution on health expenditure without controlling other variables. The results show a positive impact of air pollution on health expenditure in the studied countries. It shows that one percent increase in PM_2.5_ would increase government health expenditure by 0.25 percent (0.15 and 0.11 for private and total health expenditures, respectively). Air pollution is not the only environmental factor that affects health expenditure; hence, we control for temperature in column 2. The coefficient for temperature is positive and statistically significant explaining temperature as an important determinant of health expenditure in developing countries. The results verify that temperature if increased by one percent would increase the government health expenditure by 0.13 percent (0.10 and 0.12 for private and total health expenditures, respectively).

Health expenditure is not only influenced by environmental factors, but also through other variables such as income, population growth and education. Thus, controlling for these variables, the results suggest that a one percent increase in EDU and income will lead to increased health expenditures by 0.2, 0.08 and 0.07 percent, and 0.62, 0.71 and 0.81 for government, private and total health expenditures, respectively. However, in the case of pop, the results suggest a negative impact with a one increase in population, the health expenditure decreases by 0.18 percent for government health expenditure, while it is positive (0.06 and 0.03) in the case of private and total health expenditure.

An important point in all the specifications is the consistent positive value of PM_2.5_ for all the three regressions. Most of these values are significant at five percent with few exceptions. When we controlled for the effects of all the control variables, the coefficient values for PM_2.5_ are 0.14, 0.10 and 0.07 for government, private and total health expenditures, respectively. Similar results can be seen for the second variable of our interest, i.e., temperature, where most of the results are significant with the exception of one. When we control for the effects of all the control variables, i.e., full models for each type of health expenditures, the coefficient values for temperature were 0.11, 0.10 and 0.12 for government, private and total health expenditures, respectively. The results show a strong and significant relationship of current health expenditure with the previous year health expenditures. Education and population coefficients are significant in most of the cases, while per capita GDP and lag values of the dependent variable were also significant.

To check the robustness of our results, we divided the data into two groups, i.e., with one group consisting of a higher income than the other. This is to check for the common effect that could creep in as we regress all the data. We divided the countries into groups based on 2008, as this is the mid-year. The higher-income countries include Nicaragua, Mauritania, Nigeria, Uzbekistan, Bolivia, Philippines, Bhutan, Morocco, El Salvador, Angola, Sri Lanka, Mongolia, Egypt, Ukraine, Tunisia and Algeria, and this group is named group A. The lower income group consists of Malawi, Zimbabwe, Tanzania, Nepal, Kenya, Bangladesh, Cambodia, Benin, Senegal, Cameron, Myanmar, Ghana, Zambia, India, Vietnam, Honduras and Pakistan, and this group is named group B. Comparative results of both the groups for government, private and total health expenditures are given in Table 5:

The results show greater impact of PM_2.5_ and temperature on government health expenditure in higher-income countries as compared to lower-income countries, while for other variables, results are largely the same. In the case of private health expenditures, results show a higher impact of PM_2.5_ and temperature for higher-income countries; however, the difference between the two is less than the case of government health expenditure. In the case of total health expenditure, the difference of the impact of PM_2.5_ and temperature between higher and lower-income countries is again similar to the government health expenditure. In all cases, the impact of other controlling variables is more or less the same. With a few exceptions, most of the variables were statistically significant. One can infer from the above results that as countries grow economically, the impact of ambient pollution and temperature seems to be higher on various measures of health expenditures.

## 5. Discussion

The study analyzed the relationship between environmental quality (air pollution and temperature) and health expenditure in thirty-three developing countries. Results suggest that the higher the air pollution in an economy, the higher the healthcare expenditures. Our results are consistent with [24,26,33,40,42]. The increase in economic activities to achieve higher economic growth is associated with deterioration in environmental quality in terms of the higher usage of non-renewable resources, air pollution, global warming and loss of environmental habitats [47,48]. Thus, the increase in air pollution and the effects of climate change in these developing countries with limited resources are seriously damaging their health care systems. As well as healthcare systems, the air pollution affects the labor market by reducing the labor supply. The negative impact of air pollution on labor’s health causing them to reduce their supply or to opt for work that has little impact on their health [7,49]. Thus, air pollution not only affects the economy through an increase in health expenditure, but also the labor supply and their productivity.

Our result shows that the impact of air pollution is higher for government compared to private health expenditures which suggests that governments spend more in developing countries suggesting that governments have more of a role in providing health facilities. It may further suggest that the lower income level of people in developing countries might prevent them from spending it on their health.

Pollutants like PM_2.5_ are tiny in nature and go through the body’s defenses, penetrating deep into the respiratory and circulatory system, damaging the lungs, brain and heart [4]. Air pollution can also trigger asthma, childhood cancer and cardiovascular diseases later in life [50]. Pregnant women exposed to higher levels of air pollution are most likely to give birth prematurely or to low-birth-weight infants [4,50]. Air pollution affects people of all ages but especially children who are more susceptible during fetal and at the early years of their development when the brain and lungs are still maturing [2,51]. Ref. [52] noted that health spending is two to three times more sensitive to air pollution in children’s hospitals indicating the vulnerability of children to air pollution. They found a significant impact of air pollution on health expenditure, as health expenditure increases sharply during two to three months post exposure to air pollution, showing the persistent impact of air pollution. Therefore, higher pollution levels might put more pressure on the limited resources causing severe budget deficits for these developing countries.

Our results also show a positive relationship between temperature and health expenditure in the studied countries. Our results are consistent with the previous studies showing a positive association between temperature and health expenditure [16,40,53]. Vector borne diseases are transmitted by disease vectors such as mosquitoes, fleas and ticks. Infectious pathogens such as viruses, bacteria and protozoa can be transmitted from animals to people via these vectors. Temperature, precipitation and extreme events expand the geographic range of diseases conveyed by these vectors, thus causing illnesses, and hence burdening the healthcare facilities.

Moreover, the extreme hot weather can lead to heat stroke and dehydration, as well as cardiovascular, respiratory and cerebrovascular diseases [54]. The climate change may interact with the air quality as well, i.e., changes in the climate affect the air we breathe both indoors and outdoors. Warmer temperatures and shifting weather patterns can worsen air quality, which can lead to asthma attacks and other respiratory and cardiovascular health effects [12].

The changes in climate affect mostly children, aged people and people with disabilities and the poor. These health risks have economic spillovers not only on an individual’s savings and healthcare spending but also on healthcare demand at aggregate level. The climate change cost may continue to grow if proper policies are not adopted. Developing countries are striving hard for higher economic growth, which will further deteriorate the environment in these countries causing a serious burden on their health expenditures. The spread of climate-sensitive diseases will depend on both climate and non-climate factors such as socioeconomic conditions, access to health care and human responses to disease risk.

The effects of income on healthcare expenditure are positive which implies that as the economy grows and it resultantly increases the income of the government and the people, there is a likelihood that healthcare expenditure will increase. These results corroborate the studies of [30,55]. The increase in income helps the government and people to spend more on health and healthcare facilities considering health as a necessity. The results also show a positive effect of education on healthcare spending as education promotes a sense of responsibility among the people. Educated people will demand and avail more health facilities as compared to less educated people [56], while population has a positive effect on healthcare spending except government health expenditures. Highly populated areas have higher chances of outbreak of diseases forcing people to spend more on their health [4,56].

Although this study tried to cover the most important factors affecting health care expenditure, results should be interpreted cautiously. This is because we have included PM_2.5_ as a proxy for air pollution, while studies have included other variables such as sulfur dioxide, carbon dioxide and so forth. Although such variables do explain the level of air pollution, a more comprehensive measure including all or most of the important pollutants could be used to analyze air pollution impact. This study focused on the contemporaneous impact of environmental factors. Future studies may look into the long lasting impact of environmental factors. Moreover, other socioeconomic variables explaining socioeconomic conditions such as land use, level of urbanization, poverty and social class differences may portray a better picture.

## 6. Conclusions

The aim of the study was to contribute to the health, environmental and ecological literature which has labeled the key factors of healthcare expenditures. The novelty of this study is that we extended the model by introducing the key environmental variables in a cross-country trajectory. The study empirically examined the impact of environmental quality on healthcare expenditure in thirty-three developing countries using the dataset from 2000 to 2018. The environmental quality was measured through air pollution (PM_2.5_) and average temperature. We employed generalized method of moments (GMM) for estimating our model. The study found a positive impact of both air pollution and temperature on healthcare expenditure in the studied countries. However, the impact of environmental variables is highest for government health expenditure followed by private and total health expenditure, respectively. Moreover, we divided the countries into higher and lower income groups. Results suggest that higher-income countries are more affected by environmental variables as compared to lower-income countries. It suggests that environmental degradation caused by increased economic growth and other associated factors are responsible for higher national and private healthcare expenditure in these countries. The higher impact of such variables on government health expenditures suggests greater role of government thus suggesting an active public policy while designing the healthcare system in developing countries.

Our results further reveal that income, education and population density also have a positive and significant impact on healthcare expenditure which highlights the importance of these factors in affecting healthcare expenditures. The increased healthcare expenditure due to increased air pollution and climate change necessitate for an efficient, reliable, affordable and modern energy policy by emphasizing the use of clean and renewable energy in these countries that ensure better health for the masses. Furthermore, a smart and sustainable environmentally friendly economic growth policy is necessary to ensure better health in developing countries.

## Figures and Tables

**Table 1:** Descriptive Statistics.

Variable	Mean	Std. Dev.	Min.	Max.
Total H.E	202.3492	168.8196	12.96985	1012.947
Govt. H.E	102.1322	110.6483	2.752675	723.3528
Pvt. H.E	100.217	72.53725	3.753699	373.0385
Temp	22.19348	6.066826	−0.75833	29.175
PM_2.5_	40.17425	19.99682	11.09962	100.7844
Edu.	58.10093	23.33788	12.38947	98.3254
Pop.	7.42 × 10^7^	2.05 × 10^8^	591,014	1.34 × 10^9^
GDP	4546.763	2814.33	644.6779	13,500.04

Note: Total H.E. denotes total health expenditure, Govt. H.E. represents government health expenditure, Pvt. H.E. measures the private health expenditure, Temp is used for average temperature, PM_2.5_ represents particulate matter, Edu. denotes secondary education level as percentage of gross school enrollment, Pop. denotes population density and GDP is used for per capita GDP. We used data for 33 developing countries including, Algeria, Angola, Bangladesh, Benin, Bhutan, Bolivia, Cambodia, Cameron, Egypt, El Salvador, Ghana, Honduras, India, Kenya, Malawi, Mauritania, Mongolia, Morocco, Myanmar, Nepal, Nicaragua, Nigeria, Pakistan, the Philippines, Senegal, Sri Lanka, Tanzania, Tunisia, Ukraine, Uzbekistan, Vietnam, Zambia and Zimbabwe.

**Table 2:** Correlation among Dependent Variables.

Correlation Coefficients	Total H.E. (−1)	Govt. H.E. (−1)	Pvt. H.E. (−1)
Total H.E.	0.9024		
Govt. H.E.		0.9069	
Pvt. H.E.			0.9057

Source: Authors’ calculations.

**Table 3:** Results (Panel Fixed Effect Model).

Variable		Govt. H.E.					Private H.E.					Total H.E.			
	1	2	3	4	5	1	2	3	4	5	1	2	3	4	5
PM_2.5_	0.11 (0.03)	0.14 (0.04)	0.14 (0.04)	0.09 * (0.06)	0.06 (0.05)	0.08 (0.02)	0.10 * (0.04)	0.11 (0.04)	0.08 (0.04)	0.07 * (0.03)	0.21 (0.05)	0.19 * (0.04)	0.17 ** (0.04)	0.12 (0.03)	0.09 (0.03)
Temp		0.08 (0.03)	0.10 * (0.04)	0.13 * (0.05)	0.10 (0.03)		0.07 ** (0.01)	0.08 (0.02)	0.09 * (0.04)	0.12 (0.04)		0.08 ** (0.03)	0.09 (0.05)	0.10 (0.04)	0.11 (0.05)
Edu			0.31 * (0.11)	0.25 (0.12)	0.22 (0.15)			0.04 ** (0.03)	0.16 (0.04)	0.12 ** (0.05)			0.06 * (0.01)	0.07 (0.02)	0.10 ** (0.04)
Pop				0.14 (0.04)	0.14 ** (0.07)				0.05 (0.01)	0.11 ** (0.04)				0.04 * (0.02)	0.06 ** (0.03)
GDP					0.74 (0.15)					0.83 * (0.15)					0.91 ** (0.21)
R-Sq	0.45	0.64	0.67	0.72	0.88	0.42	0.49	0.56	0.67	0.78	0.56	0.61	0.67	0.76	0.82
Hausman Test					24.72 ***					19.64 ***					25.64 ***

Note: *, **, *** denotes results significance at 10, 5 and 1 percent, respectively, while values in brackets denote standard errors, whereas PM_2.5_ denotes the ambient air pollution, Temp denotes average temperature, Edu shows education level, Pop shows population density and GDP shows per capital GDP. Hausman test is reported for final specification only.

**Table 4:** Results (System GMM).

Variable		Govt. H.E.					Private H.E.					Total H.E.			
	1	2	3	4	5	1	2	3	4	5	1	2	3	4	5
PM_2.5_	0.25 ** (0.16)	0.24 ** (0.07)	0.28 * (0.09)	0.13 ** (0.05)	0.14 ** (0.05)	0.15 ** (0.07)	0.11 ** (0.04)	0.10 *** (0.01)	0.10 * (0.00)	0.10 ** (0.02)	0.11 ** (0.01)	0.09 * (0.01)	0.08 ** (0.01)	0.07 ** (0.01)	0.07 ** (0.01)
Temp		0.13 ** (0.04)	0.12 * (0.01)	0.11 ** (0.03)	0.11 ** (0.03)		0.10 ** (0.02)	0.11 (0.07)	0.10 ** (0.08)	0.10 ** (0.07)		0.12 ** (0.11)	0.14 * (0.12)	0.13 ** (0.13)	0.12 ** (0.12)
Edu			0.20 * (0.05)	0.18 (0.07)	0.17 * (0.05)			0.08 ** (0.03)	0.16 (0.09)	0.17 ** (0.08)			0.07 * (0.02)	0.08 (0.01)	0.08 ** (0.03)
Pop				−0.18 (0.07)	0.19 ** (0.09)				0.06 * (0.03)	0.19 ** (0.09)				0.03 * (0.01)	0.03 ** (0.01)
GDP					0.62 ** (0.01)					0.71 * (0.01)					0.81 ** (0.01)
Lag	0.79 ** (0.01)	0.79 ** (0.01)	0.78 ** (0.01)	0.80 * (0.01)	0.79 ** (0.01)	0.99 ** (0.01)	0.98 * (0.01)	0.89 ** (0.01)	0.82 ** (0.01)	0.79 * (0.01)	0.95 ** (0.01)	0.91 * (0.01)	0.86 * (0.01)	0.87 ** (0.01)	0.85 ** (0.01)
AR (1)	0.008	0.007	0.006	0.006	0.007	0.005	0.005	0.005	0.004	0.007	0.007	0.004	0.005	0.004	0.0001
AR (2)	0.212	0.199	0.187	0.184	0.162	0.356	0.344	0.332	0.321	0.281	0.257	0.241	0.210	0.209	0.201
Hansen (OIR)	0.971	0.953	0.934	0.922	0.913	0.967	0.960	0.945	0.935	0.931	0.945	0.931	0.922	0.918	0.905

*, **, *** denotes results significance at 10, 5 and 1 percent, respectively, while values in brackets denote standard errors, whereas PM_2.5_ denotes the ambient air pollution, Temp denotes average temperature, Edu shows education level, Pop shows population density, GDP shows per capital GDP and Lag shows the dependent variable’s lagged values.

**Table 5:** Comparative Results of Higher-& Lower-Income Countries.

Variable	Govt. Heath Exp.		Private Health Exp.		Total Health Exp.	
	Group A	Group B	Group A	Group B	Group A	Group B
PM_2.5_	0.31 (0.05)	0.11 (0.05)	0.23 (0.02)	0.12 (0.02)	0.26 (0.01)	0.07 (0.01)
Temp	0.20 (0.03)	0.10 (0.03)	0.13 (0.07)	0.12 (0.07)	0.15 (0.12)	0.11 (0.12)
Edu	0.18 (0.05)	0.13 (0.05)	0.16 (0.08)	0.10 (0.08)	0.10 (0.03)	0.07 (0.03)
Pop	0.11 (0.09)	0.15 (0.09)	0.12 (0.09)	0.12 (0.09)	0.08 (0.01)	0.03 (0.01)
GDP	0.79 (0.01)	0.69 (0.01)	0.71 (0.01)	0.78 (0.01)	0.69 (0.01)	0.74 (0.01)
Lag	0.78 (0.01)	0.78 (0.01)	0.79 (0.01)	0.77 (0.01)	0.81 (0.01)	0.81 (0.01)
AR (1)	0.002	0.001	0.003	0.009	0.002	0.0001
AR (2)	0.385	0.151	0.275	0.411	0.390	0.225
Hansen (OIR)	0.913	0.919	0.927	0.922	0.903	0.900

## Data Availability

Not applicable.
